# Extracellular vesicles delivering TIMP-2 modulate MMP-1, MMP-2, and MMP-9 expression in human lung adenocarcinoma A549 cells

**DOI:** 10.3389/fphar.2026.1784404

**Published:** 2026-02-26

**Authors:** Agnieszka Stawarska, Magdalena Bamburowicz-Klimkowska, Maciej Małecki, Anna M. Nowicka, Żaneta Słyk, Agata Kowalczyk, Alicja Targonska, Ireneusz P. Grudzinski

**Affiliations:** 1 Department of Toxicology and Food Science, Faculty of Pharmacy, Medical University of Warsaw, Warsaw, Poland; 2 Department of Applied Pharmacy, Faculty of Pharmacy, Medical University of Warsaw, Warsaw, Poland; 3 Faculty of Chemistry, University of Warsaw, Warsaw, Poland; 4 Laboratory of Molecular Bases of Ageing, Nencki Institute of Experimental Biology, Polish Academy of Sciences, Warsaw, Poland

**Keywords:** electroporation, extracellular vesicles, gene encapsulation, lung cancer cells, matrix metalloproteinases, tissue inhibitor of metalloproteinase

## Abstract

**Background/Objectives:**

Extracellular vesicles (EVs) carrying therapeutic cargos represent a promising strategy for cancer treatment by enabling the targeted delivery of genetic material directly to cancer cells. This study aimed to evaluate the effect of EVs loaded with the TIMP-2 gene on the expression of matrix metalloproteinases (MMPs 1, 2, and 9) in lung cancer cells (A549).

**Methods:**

EVs derived from A549 cells were isolated by gradient centrifugation and ultracentrifugation. The coding sequence for TIMP-2 (tissue inhibitor of metalloproteinases 2) was amplified by PCR using cDNA derived from HUVEC cells. As-constructed plasmid (pTIMP-2) was introduced into the EVs by electroporation, and then the pTIMP-2-implanted EVs were subjected to PCR and NTA analysis. Additionally, the activity of MMP-1, MMP-2, and MMP-9 was determined by voltammetry in intact A549 cells and in A549 culture media.

**Results:**

Electroporation was found to demonstrate a good potential as an exogenous technique for uploading plasmid DNA into EVs. The results demonstrated that the as-uploaded EVs carrying the pTIMP-2 gene cargo do not broadly alter the overall balance of MMP-1 in pristine A549 cells. However, pTIMP-2-loaded EVs significantly modulate MMP-2 and MMP-9 expression in these cells, highlighting their potential as biological therapeutic moieties.

**Conclusion:**

Our findings suggest a rational approach for exploring EV-based gene transfer targeting MMPs in lung cancer.

## 1 Introduction

Extracellular vesicles (EVs) are naturally occurring, nanosized membrane-bound particles secreted by a variety of cells throughout the body. They play an essential role in intercellular communication by transferring bioactive molecules between cells (Xie et al., 2022). EVs are increasingly recognized as promising vehicles for gene delivery due to their inherent biocompatibility, low immunogenicity, and ability to traverse physiological barriers such as the blood-brain barrier. EVs provide a natural, efficient delivery system that protects genetic material from degradation, enhances its uptake by target cells, and enables targeted delivery, improving therapeutic outcomes and providing safe therapies. Loading therapeutic genes into EVs enables the delivery of functional DNA, RNA, or other nucleic acid constructs directly to target cells, where they can restore, replace, or modulate gene expression. Approaches for loading genes into EVs can be broadly divided into endogenous and exogenous strategies (Han et al., 2021). Endogenous methods rely on engineering EV-producing cells to naturally package genetic material. Donor cells can be transfected with plasmid DNA constructs, resulting in EVs loaded with corresponding mRNA or DNA cargos. Fusing EV surface proteins, such as CD63 or CD9, with RNA- or DNA-binding domains enhances cargo sorting into EVs (Sun et al., 2023). Advanced systems combine protein fusions and specific RNA motifs to selectively load mRNAs into EVs while boosting vesicle release and delivery efficiency (Corrado et al., 2021). The exogenous approach involves direct loading of biomolecules into isolated EVs via electroporation, sonication, incubation, freeze-thaw, and/or transfection (Han et al., 2021). Electroporation uses electric pulses to transiently permeabilize EV membranes, allowing plasmid DNA moieties entry, although smaller DNA fragments are incorporated more efficiently than larger plasmids (Lichá et al., 2022). Chemical permeabilization with agents such as calcium chloride or saponins can also destabilize EV membranes, facilitating nucleic acid loading (Han et al., 2021). Freeze-thaw cycles temporarily disrupt EV membranes, enabling oligonucleotide entry. Fusion with liposomes creates hybrid vesicles capable of carrying DNA or RNA, while hybrid EVs generated via freeze-thawing with liposomes improve loading and uptake of larger genetic constructs, including CRISPR/Cas9 components (Oshchepkova et al., 2024).

DNA-based therapy involves using genetic material to directly modify or replace defective genes in patients, offering promising strategies for treating various genetic disorders, including mitochondrial-associated diseases (Di Donfrancesco et al., 2022), inherited genetic disorders (Wu et al., 2024), and potentially even cancers (Das et al., 2015). Unlike RNA-based therapies, which primarily modulate gene expression, DNA-based therapies aim to replace, repair, or edit specific genes to restore normal function. The use of DNA in gene therapy, especially in treating genetic and mitochondrial diseases, holds significant promise for correcting genetic defects at their source. By delivering functional copies of genes or employing gene-editing techniques, DNA-based therapies have the potential to transform the treatment of inherited disorders and cancers (Wallen et al., 2023; Kumar et al., 2024). Although there are challenges in efficient delivery, safety, and cost, advances in technology, including the use of EVs for DNA delivery, may provide more effective and less invasive ways to harness the power of DNA-based therapies in clinical settings (Schulz-Siegmund and Aigner, 2021; Oshchepkova et al., 2024). Nowadays, it is possible to load and deliver various types of RNA, including mRNA, miRNA, siRNA, shRNA, lncRNA, and others, into extracellular vesicles. Note that mRNA encapsulated in EVs offers a highly versatile and promising strategy for gene therapy (Kirian et al., 2024; Di Ianni et al., 2025) and immunotherapy (Zhang et al., 2025). By delivering functional mRNA to target cells, it is possible to correct genetic defects, replace missing enzymes, and stimulate immune responses for the treatment of a wide range of diseases, including genetic disorders, cancers, and viral infections (Cecchin et al., 2023; Gai et al., 2024; Nakagawa et al., 2026). Whereas miRNAs are potent regulators of gene expression, and their potential as therapeutic agents is increasingly being realized, particularly in the contexts of cancer and viral infections (YiRan Li et al., 2025). By loading miRNAs into EVs, it becomes possible to deliver these regulatory molecules efficiently and specifically to target cells, restoring regular gene expression, silencing oncogenic genes, or modulating immune responses (Goyal et al., 2025). siRNA is also a powerful tool for targeted gene silencing. When encapsulated in EVs, it offers a promising strategy for therapeutic gene knockdown in a variety of diseases, including cancer, genetic disorders, viral infections, and inflammatory diseases (Roerig et al., 2022; Rong et al., 2023). Likewise, shRNA offers a powerful approach for inducing long-term gene silencing and, when delivered via EVs, holds great potential for treating chronic diseases, cancer, viral infections, and genetic disorders (Rong et al., 2023). LncRNAs, in turn, play crucial roles in gene regulation, influencing both epigenetic and post-transcriptional processes. By modulating chromatin structure, transcriptional machinery, RNA stability, and translation, they are key regulators of cellular functions and are implicated in a wide range of diseases (Spanos et al., 2023). Circular RNAs are a class of non-coding RNAs that play crucial roles in modulating gene expression through miRNA sequestration and protein binding (Zhao et al., 2024). They have diverse biological functions, including regulation of cell differentiation, proliferation, and apoptosis, and are implicated in a wide range of diseases, particularly cancer and cardiovascular disorders (Gu et al., 2023). Given their stability and ability to regulate gene expression in a specific and controlled manner, circRNAs offer promising avenues for therapeutic interventions (Fang et al., 2024).

Tissue inhibitors of metalloproteinases (TIMPs) are a family of proteins that regulate matrix metalloproteinases (MMPs). Among them, TIMP-2 has attracted attention for its potential role in cancer therapy, particularly for its dual function in tumor progression and metastasis (Stetler-Stevenson, 2023). While it inhibits MMPs and thus can block the remodeling of the extracellular matrix (ECM) required for tumor cell invasion, recent studies have highlighted its complex role in cancer biology, including tumor-suppressive effects (Peeney et al., 2022). TIMP-2 has been proposed as a tumor-suppressive agent because it inhibits MMPs, which are often overexpressed in tumors and facilitate tumor growth and metastasis. MMPs degrade ECM components, allowing tumor cells to invade surrounding tissues and spread to distant organs. By inhibiting MMPs, TIMP-2 can restrict tumor cell migration and invasion, thus potentially reducing metastatic potential (Baker et al., 2002; Almutairi et al., 2023). Additionally, studies have shown that overexpression of TIMP-2 in various cancer models, such as breast cancer, reduces tumor growth and metastasis (Peeney et al., 2020). In breast cancer specifically, TIMP-2-mediated inhibition of MMP-2 has been shown to reduce cancer cells’ ability to degrade the ECM, thereby limiting metastasis to the lungs and other organs (Peeney et al., 2020). Despite its potential tumor-suppressive properties, TIMP-2’s role in cancer is not straightforward. Recent research has uncovered the dual nature of TIMP-2 in the tumor microenvironment. TIMP-2, while inhibiting MMP activity, can also activate pro-angiogenic factors. For instance, TIMP-2 has been shown to interact with vascular endothelial growth factor (VEGF) and promote angiogenesis, a process essential for tumor growth and survival (Stetler-Stevenson, 2008b; Quintero-Fabián et al., 2019). TIMP-2 may promote tumor growth by enhancing tumor blood supply, providing it with the nutrients and oxygen needed to sustain its progression. The balance between its inhibitory effects on MMPs and its involvement in pro-tumorigenic signaling pathways complicates TIMP-2’s role in cancer treatments.

In this study, we investigate the regulatory effects of extracellular vesicles encapsulating TIMP-2 gene cargos on the expression and activity of matrix metalloproteinases (MMP-1, MMP-2, and MMP-9) in human lung adenocarcinoma cells (A549). Given the pivotal role of MMPs in extracellular matrix remodeling, tumor invasion, and metastasis, we aim to elucidate whether EV-mediated delivery of TIMP-2 can attenuate MMP-associated proteolytic activity and consequently suppress the invasive potential of lung cancer cells. Through a combination of molecular and biochemical analyses, this study seeks to provide mechanistic insight into the therapeutic potential of TIMP-2–enriched EVs as modulators of tumor microenvironment dynamics and metastatic progression.

## 2 Materials and methods

### 2.1 Isolation and characterization of extracellular vesicles

Human adenocarcinomic alveolar basal epithelial cell line - A549 (ATCC CCL-185) was obtained from American Type Culture Collection (ATCC, Manassas, VA, United States). The A549 cells were grown as an adherent monolayer in F-12K medium (Kaighn’s modification of Ham’s F-12 medium; Gibco, Paisley, supplemented with 10% fetal bovine serum (FBS; Gibco, Paisley, United Kingdom) and antibiotics (streptomycin, 50 µg·mL^−1^; amphotericin B, 1.25 µg·mL^−1^; gentamicin, 50 µg·mL^−1^; penicillin, 50 µg·mL^−1^) (Gibco, Paisley, United Kingdom). Before EV isolation, the standard media was replaced with a 10% exosome-depleted FBS (One ShotTM format, Gibco, Paisley, United Kingdom), and A549 cells were incubated for a further 3 days in T225 culture flasks. Cell culture media were harvested from A549 cells, and extracellular vesicles were isolated using gradient centrifugation and ultracentrifugation as described in detail elsewhere (Ruzycka-Ayoush et al., 2023). In protein analysis, the Pierce BCA Protein Assay Kit (Thermo Fisher Scientific™, United States) was used according to the manufacturer’s instructions to determine protein concentration. Briefly, 20 μL of each standard and sample were added to a 96-well plate. Then, each well was added 200 μL of working reagent (mixed 50 parts of BCA Reagent A with 1 part of BCA Reagent B). The 96-well plate was covered and incubated at 37 °C for 30 min. The absorbance was measured at 562 nm on a spectrophotometric multi-well plate reader. Particle size and distribution analysis of extracellular vesicles were performed using nanoparticle tracking analysis (NTA) with NanoSight NS300 (Malvern Panalytical Ltd., United Kingdom) equipped with a 488 nm blue laser (Ruzycka-Ayoush et al., 2023). Specific biomarkers of extracellular vesicles, such as the tetraspanins CD9, CD63, and CD81, were assayed using surface plasmon resonance (SPR) as developed in-house and described previously (Kowalczyk et al., 2023). Please note that this method was validated against the Western blot assay, as routinely used to assess EV characteristics in our laboratories (Ruzycka-Ayoush et al., 2023). The stability of A549-derived EVs was evaluated based on their physicochemical parameters, including size distribution and particle concentration, using nanoparticle tracking analysis (NTA) and dynamic light scattering (DLS) to measure surface zeta potential (ZP). The results obtained have already been described in a previous publication (Ruzycka-Ayoush et al., 2023).

### 2.2 Construction of the pTIMP2 vector

The TIMP-2 coding sequence was amplified by PCR from cDNA using HUVEC cells. Primers were designed with restriction sites for EcoRI and XhoI (T2-1: 5′AAG​AAT​TCC​TGC​AGC​TGC​TCC​CCG​G3'; T2-2: 5′AAC​TCG​AGC​CAC​AGG​GGC​GTT​GGA​G3′). The PCR product was separated by electrophoresis, isolated from an agarose gel using the QIAquick Gel Extraction Kit (QIAGEN), digested with EcoRI/XhoI (Promega) for 1 h at 37 °C, and purified using the Cleanup Kit (QIAGEN). The pSECTag2B vector was also digested with EcoRI/XhoI, separated by electrophoresis, and isolated from the gel using a QIAquick Gel Extraction Kit (QIAGEN). Ligation was carried out for 16 h at 16 °C using T4 DNA ligase (Promega). The ligation products were transformed into *E. coli* bacteria (DH5α), and clone selection was performed on ampicillin-containing agar plates. The grown clones were passaged into liquid LB medium (LB Broth, Sigma) with ampicillin and cultured overnight at 37 °C. Plasmids were isolated from the clones, which were then subjected to restriction analysis, PCR, and sequencing. The studies confirmed the presence of the correct TIMP-2 gene sequence in the pSECTag2B vector. A plasmid expression vector containing the TIMP-2 gene was obtained.

### 2.3 pTIMP-2 uploaded into extracellular vesicles

Extracellular vesicles derived from A549 cells were suspended in phosphate-buffered saline (PBS, pH 7.2) to a protein concentration of 0.4 mg mL^−1^, which corresponds to approximately 5.8 × 10^10^ EVs mL^−1^. The series of EVs is characterized by a ZP of −24 mV and a PDI of 0.115. The TIMP-2 gene was dissolved in PBS to a concentration of 1 µg µL^−1^. A 100 µL aliquot of the exosome suspension was measured, and 5 µL of the TIMP-2 gene solution was added. The mixture was subjected to optimized electroporation parameters: 250 V, one pulse, 25 ms, using a square wave pulse generator (BTX ECM 830, Waterbeach, Cambridge, United Kingdom), and then incubated for 18 h at room temperature to facilitate cellular uptake of pTIMP-2. Although PBS is a salt-containing buffer, electroporation was performed using current-limiting devices and optimized pulse settings (short pulses, minimum effective voltage) to avoid arcing and overheating. Samples were checked for air bubbles and transferred to cuvettes with parameters appropriate for the applied field. The samples were then centrifuged at 100,000 g for 90 min at 4 °C to pellet the cells. The supernatant was discarded, and the pellet was washed twice with PBS, with ultracentrifugation at 100,000 g for 90 min at 4 °C after each wash to remove excess pTIMP2 before further processing. The cell pellet was resuspended in 200 µL of PBS. A 50 µL aliquot of the cell suspension was used to determine the pTIMP-2 vector copy number. A 50 µL aliquot of the cell suspension was also subjected to nanoparticle tracking analysis (NTA) to determine the size distribution and concentration of TIMP-2-loaded EVs.

### 2.4 Evaluation of pTIMP-2 vector copy copies

Quantitative PCR (qPCR) was performed to evaluate the copy number of the TIMP-2 gene. A standard curve was generated using serial dilutions of plasmid DNA (pTIMP-2). The preparation of the standard curve began with the calculation of the number of plasmid particles in 1.0 µL of pTIMP-2 DNA. This calculation considered the plasmid’s concentration, size, and molar mass. Based on the calculated number of gene copies per microliter, a series of dilutions ranging from 10^9^ to 10^0^ gene copies per µL was prepared. The standard curve generated from these dilutions was used to determine reaction efficiency, which ranged from 95% to 105%. The analysis was performed using the TaqMan® Gene Expression Assay (Assay ID: Hs00234278_m1). Each qPCR reaction had a total volume of 10 µL and was carried out under the following thermal cycling conditions: 50 °C for 2 min, 95 °C for 10 min, followed by 40 cycles of 95 °C for 15 s and 60 °C for 60 s. Real-time PCR was performed using the StepOnePlus™ Real-Time PCR System (Applied Biosystems, Thermo Fisher Scientific). Each reaction was conducted in triplicate.

### 2.5 Cell studies and metalloproteinase analysis

To assess the effect of the pTIMP-2 loaded EVs on MMPs to A549 cells, the cells were seeded in 24-well plates at 5.6 × 10^4^ cells per well suspended in 500 µL of F-12 K medium (Kaighn’s modification of Ham’s F-12 medium (Gibco, Paisley), supplemented with 10% fetal bovine serum (FBS; Gibco, Paisley, United Kingdom), streptomycin, 50 μg⋅mL^−1^, amphotericin B, 1.25 μg⋅mL^−1^, gentamicin, 50 μg⋅mL^−1^, penicillin, 50 μg⋅mL^−1^ (all from GIBCO, Paisley, United Kingdom). The cells were routinely cultured at 37 °C in a humidified atmosphere with 5% CO2. After 24 h of incubation, pTIMP-2 loaded EVs were added to the wells at the following concentrations: 10 × 10^6^, 10 × 10^8^, 10 × 10^10^ EVs mL^−1^ and plasmid-free EVs in concentration 10 × 10^10^ particles mL^−1^. The A549 cells treated with no pTIMP-2-loaded EVs served as the negative control (NC). The prepared plates were incubated for 24 or 48 h, after which the medium was collected into Eppendorf tubes, and the cells were lysed with IP Lysis Buffer (Pierce™ IP Lysis Buffer, Thermo Scientific). The digested cells were collected in Eppendorf tubes, and the plates were carefully examined under a microscope. Cell lysates and collected medium were analyzed for MMP-1, MMP-2, and MMP-9 concentrations using the nanosensor, as previously developed in our laboratory (Winkler et al., 2024). The MMPs recognition process was based on the proteolysis of the receptor (dipeptide functionalized with redox probe: Cys-Gly-Ile-MB (MMP-1), Cys-Gly-Leu-AQ (MMP-2), and Cys-Gly-Met-Fc (MMP-9)). The hydrolysis of the appropriate peptide bond removes the fragment of the receptor with the redox probe, and, as a consequence, the current signal intensity decreases. The sensor used in the study exhibited a wide linear range from 1.0·10^−9^ to 1.0 mg·L^−1^ and low limit of quantification values of 27.7, 31.5, and 45.2 fg·L^−1^ for MMP-9, MMP-1, and MMP-2, respectively. All voltammetric measurements were performed using an Autolab potentiostat PGSTAT 30 in a three-electrode system consisting of a working electrode (gold disc electrode, f = 1.6 mm), a reference electrode (Ag/AgCl/3 M KCl), and an auxiliary electrode (platinum plate with an area of at least 1 cm^2^). Before the experiments, the surface of the working electrode was mechanically polished on a wet pad with the addition of alumina slurry (1 mm), and washed with a direct stream of ultra-pure water (Hydrolab, conductivity ∼0.056 μS⋅cm^−1^) to remove remains of Al_2_O_3_. The Au electrode purity degree was controlled by voltammetric cycling in 0.1 M H_2_SO_4_ in a potential value range of −0.3 to 1.5 to −0.3 V (vs. Ag/AgCl/3 M KCl) with a scan rate of 0.05 V⋅s^−1^, until a stable and typical for an unmodified gold electrode voltammogram was obtained.

### 2.6 Statistics

Data were analyzed using GraphPad Prism (version 10.6.0). A two-way ANOVA was performed to assess the effects of exposure duration and exposure factors on MMP-1, MMP-2, and MMP-9 concentrations in lysed cells and medium after 24 h and 48 h of exposure. Post-hoc comparisons were conducted using Tukey’s test for multiple comparisons. All data are presented as the mean ± standard deviation (SD) for n = 3. A significance level of p < 0.05 was considered statistically significant.

## 3 Results and discussion

**FIGURE 1:**
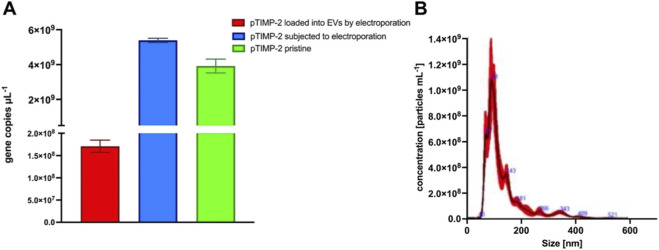
PCR and NTA analysis of EVs loaded with pTIMP-2 via electroporation. **(A)** Shows the gene copy determined by PCR for EVs loaded with pTIMP-2, non-electroporated pTIMP-2, and electroporated pTIMP-2. **(B)** Presents the corresponding NTA analysis of EVs loaded with pTIMP-2. All data are expressed as the mean ± standard deviation (SD), with *n* = 3.

Gene-loaded extracellular vesicles represent a promising strategy in cancer therapy, enabling targeted delivery of genetic material directly to tumor cells. Due to their natural biocompatibility and ability to cross biological barriers, they can enhance treatment efficacy while minimizing adverse side effects. Among recently developed gene-loaded extracellular vesicles (Gong et al., 2025), the as-developed TIPM-2-loaded EVs represent a novel, previously unheard-of therapeutic approach that can modulate MMP activity in lung cancer cells. In the study, we loaded plasmid DNA (TIMP-2) into extracellular vesicles derived from lung cancer cells (A549). The EVs uploaded with this metalloproteinase inhibitor were subjected to PCR and NTA analysis, and the results are shown in Figure 1. The loading efficiency was calculated based on the gene copy obtained in PCR for the mixture of non-electroporated EVs with pTIMP-2 (Figure 1A). Note that the loading efficiency of plasmid DNA (pDNA) into EVs is a critical parameter for optimizing the delivery of genetic material for therapeutic applications. The efficiency of loading depends on several factors, including the method of EV isolation, the electroporation conditions, and the characteristics of the pDNA cargo itself (Lamichhane et al., 2015; Han et al., 2021). Among various strategies for loading pDNA into EVs, electroporation is one of the most commonly used due to its efficiency in transferring genetic material into the vesicles (Lamichhane et al., 2015; Lichá et al., 2022; de Caro et al., 2023). During electroporation, an electric field is applied to induce transient membrane pores in the EVs, allowing the entry of molecules, such as plasmid DNA. However, achieving a high loading efficiency while minimizing vesicle damage is a significant challenge in this process. In our study, electroporation was performed with a single 25-ms pulse at 250 V. In this study, the PCR analysis confirmed that the loaded plasmid DNA was successfully transferred into EVs, although the overall efficiency remained modest. However, this value is consistent with previous reports, which show that nucleic acid electroporation typically yields loading efficiencies of 0.5%–4% (Kooijmans et al., 2013; Liang et al., 2020). The relatively low efficiency is partly due to the size and structural integrity of EVs, which can limit the amount of plasmid DNA that can be successfully encapsulated. Several factors contribute to the observed loading efficiency of plasmid DNA into EVs, including electroporation parameters, plasmid DNA properties, EV characteristics, and post-loading analysis (Lamichhane et al., 2015; Orefice, 2020). The voltage, pulse duration, and number of pulses all affect the efficiency of DNA loading. While higher voltage or longer pulse duration can increase loading efficiency, they also increase the risk of damaging EV membranes, leading to a loss of vesicle integrity (Pomatto et al., 2019). In our study, a 25-ms pulse at 250 V was chosen to balance effective DNA loading with minimal vesicle disruption. The size and conformation of the plasmid DNA can also influence its ability to be loaded into EVs. Larger plasmids may be more challenging to incorporate into the vesicles, while smaller plasmids or linearized forms might load more efficiently. The use of supercoiled plasmids or linearized DNA could improve the loading efficiency (Ohse et al., 1995; Lamichhane et al., 2015). The size and surface properties of EVs also play a critical role. Smaller vesicles, for instance, might have a greater surface area-to-volume ratio, potentially allowing for higher DNA uptake. Moreover, the lipid composition of the EV membrane can affect its permeability, thereby influencing the efficiency with which plasmid DNA is incorporated (Malkin and Bratman, 2020). NTA analysis of these particles revealed a size distribution consistent with the expected profile for EVs. The concentration of EVs was measured at 4.16 × 10^9^ per mL, further confirming the presence of a substantial population of extracellular vesicles (Figure 1B). These results indicate a heterogeneous distribution of EVs, with a predominant size range of 69–210 nm. The mean particle size was 125.6 nm, with a mode of 87.8 nm. The standard deviation (SD) was 70.6 nm, indicating some heterogeneity in particle size (Figure 1B). The observed heterogeneity in particle size could be attributed to the electroporation technique, in which variations in vesicle membrane permeability and the efficiency of plasmid DNA encapsulation resulted in a diverse population of EVs with different sizes.

**FIGURE 2:**
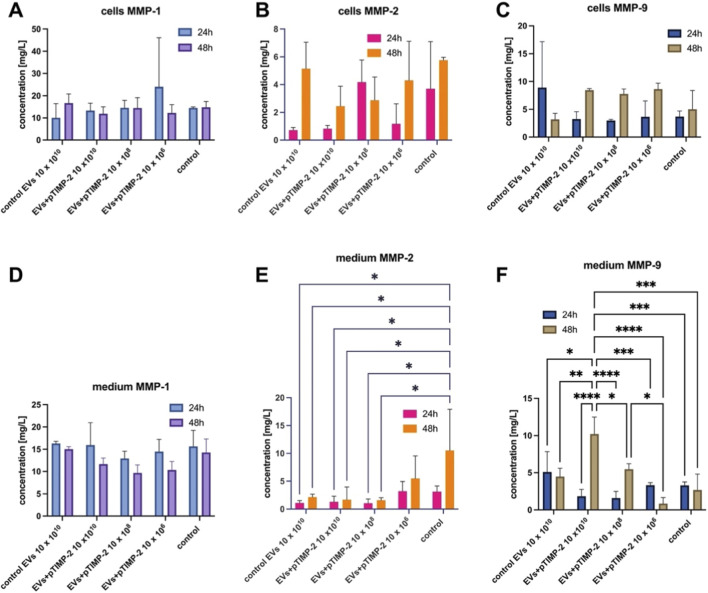
Effect of various concentrations of TIMP-2-loaded EVs on MMP-1, MMP-2, and MMP-9 levels in lung cancer cells. MMP levels were measured in both cell lysates **(A–C)** and medium **(D–F)** collected after 24 and 48 h of exposure. A two-way ANOVA was performed to assess the effects of the exposure duration and exposure factors on the MMP-1, MMP-2, and MMP-9 concentration in lysed cells and medium after 24 h and 48 h of exposure. *Post-hoc* comparisons were conducted using Tukey’s test for multiple comparisons. All data are presented as the mean ± standard deviation (SD) for *n* = 3. A significance level of *p* = 0.0109 (*), *p* = 0.0035 (**), *p* = 0.0001 (***) and *p* < 0.0001 (****) were considered statistically significant.

The use of extracellular vesicles as delivery vehicles for therapeutic cargos has been explored in several studies (Couch et al., 2021; Lyu et al., 2023; Wang et al., 2025). However, there are currently no publications describing the use of EVs loaded with TIMP-2 cargos as a potential gene therapy strategy in cancer treatment. While TIMP-2 has been widely studied as an endogenous inhibitor of MMPs, with a central role in regulating extracellular matrix remodeling and tumor invasion, and EVs have been increasingly explored as delivery vehicles for therapeutic molecules, the specific combination of TIMP-2 delivery via EVs has not been reported in the scientific literature to date. This highlights a gap in current research and suggests a possible novel direction for future studies in cancer therapy. The pTIMP-2-loaded EVs prepared in this manner were then investigated for their effects on matrix metalloproteinases 1, 2, and 9 in lung cancer cells. The cells were exposed to various concentrations of pTIMP-2-loaded-EVs (10 × 10^6^, 10 × 10^8^, 10 × 10^10^ per mL), and the levels of MMP-1, MMP-2, and MMP-9 were measured in cell lysates and in conditioned medium collected after 24 and 48 h of exposure (Figure 2). This approach allowed for the assessment of the temporal changes in MMPs expression and the potential impact of TIMP-2-loaded EVs on the regulation of matrix metalloproteinases in lung cancer cells. The statistical analysis showed that both 24-h and 48-h exposure did not result in any changes in the levels of the analyzed MMPs in the cell lysates (Figures 2A–C). Additionally, no statistically significant changes in MMP-1 concentration in the medium were observed (Figure 2D). However, significant differences in MMP-2 and MMP-9 levels were observed after 48 h of exposure to EVs (Figures 2E,F). In the case of MMP-2, concentration-dependent differences in the levels of this metalloproteinase were observed, compared to the medium collected from non-exposed cells after 48 h. An apparent inhibition of MMP-2 expression is observed (Figure 2E). The results suggest that exposure to TIMP-2-loaded EVs can alter MMP expression in lung cancer cells. Notably, the lack of significant changes in MMP-1 levels in both cell lysates and conditioned medium at 24 and 48 h indicates that the TIMP2-loaded EVs may not directly affect MMP-1 regulation in this experimental setup. This could imply that the TIMP-2 cargo specifically affects the balance of MMPs through mechanisms that are more selective to specific proteases, rather than a global effect on all MMPs. Interestingly, the significant differences observed in MMP-2 levels after 48 h of exposure to EVs suggest that TIMP-2-loaded EVs specifically modulate MMP-2 expression in a concentration-dependent manner. The inhibition of MMP-2 expression is especially notable. MMP-2 plays a pivotal role in the degradation of ECM components, promoting cancer cell invasion and metastasis. Therefore, this finding suggests that TIMP-2 may act as a regulatory factor to limit MMP-2 activity, potentially inhibiting tumor progression and metastasis. The concentration-dependent effects observed may indicate a threshold at which EVs loaded with TIMP-2 can effectively modulate MMP-2 activity. Our findings on the inhibition of MMP-2 expression following exposure to TIMP-2-loaded EVs align with previous studies on TIMP-2’s role in regulating matrix metalloproteinases, particularly MMP-2, in cancer cells. As noted by Shen et al. (2010), TIMP-2 acts as a potent inhibitor of MMP-2 activity in cancer cells, thereby limiting their invasive capacity. Our observation of MMP-2 inhibition in a concentration-dependent manner further supports the notion that TIMP-2 serves as a key regulatory molecule in ECM remodeling and tumor progression, particularly in lung cancer (Yang et al., 2010). Bourboulia et al. (2011) also reported that exogenous TIMP-2 could suppress MMP-2 secretion, thereby reducing cancer cell migration *in vitro*. This study underscores the role of TIMP-2 in modulating MMP-2 activity and supports our finding that TIMP-2-loaded EVs can reduce MMP-2 levels in lung cancer cells. Importantly, the results suggest that TIMP-2-loaded EVs can deliver this regulatory effect in a targeted and efficient manner, potentially offering a novel therapeutic approach to combat cancer cell invasion and metastasis. Interestingly, despite the observed effects on MMP-2, no significant changes in MMP-1 levels were detected in either cell lysates or conditioned medium (Figure 2D). This selective regulation of MMP-2 suggests that TIMP-2 may have a specific inhibitory effect on certain MMPs, which may be attributed to differences in their regulatory pathways or interactions with TIMP-2 (Pietruszewska et al., 2016; Kaczorowska et al., 2020). This finding warrants further investigation into the specificity of TIMP-2 for different MMPs and its role in various stages of cancer progression.

In the case of MMP-9, observed differences are more complex. A concentration-dependent decrease in MMP-9 levels was observed, proportional to the level of exposure to TIMP-2-loaded EVs after 48 h. The level of MMP-9 in the medium was highest after exposure to the highest concentration of modified EVs. Compared to this level, we observed the greatest number of statistically significant decreases in MMP-9 levels (Figure 2F). The observed concentration-dependent decrease in MMP-9 levels following exposure to TIMP-2-loaded EVs, with the highest MMP-9 levels observed after exposure to the highest concentration of modified EVs, suggests a complex interaction between TIMP-2 and MMP-9 regulation. The interaction between TIMP-2 and MMP-9 is relatively weak compared to other TIMP-MMP pairs. TIMP-2 is primarily known for its dual role in regulating MMP-2; in other words, at low concentrations, it participates in the activation of pro-MMP-2 via an MT1-MMP/TIMP-2 complex, whereas at higher concentrations, it directly inhibits active MMP-2 (Murphy, 2011). In contrast, MMP-9 is preferentially regulated by TIMP-1 rather than TIMP-2. While TIMP-2 can bind to MMP-9, its affinity is low, and the inhibitory effect is limited. As a result, TIMP-2 plays only a minor role in regulating MMP-9 activity. Moreover, active MMP-9 can proteolytically cleave TIMP-2 (particularly at the C-terminal region), producing truncated fragments that lose their standard inhibitory functionality - this additional layer of interaction complicates the TIMP–MMP balance in the extracellular matrix (Coates-Park et al., 2023). On the other hand, MMP-2 can activate pro-MMP-9, the inactive form of MMP-9, through proteolytic cleavage (Toth et al., 2003). Therefore, when both MMP-2 and MMP-9 are present in the same cells, their activities may be closely linked. MMPs are part of a broader family of enzymes that often regulate one another’s activity. MMP-9 and MMP-2, for example, can activate each other in some contexts (Gonzalez-Avila et al., 2019). Moreover, cancer cells usually exhibit dysregulated ECM remodeling and may use feedback loops to regulate the production of distinct MMPs (Cox and Erler, 2011). If TIMP-2 is inhibiting MMP-2 and the cell perceives a reduction in ECM degradation, it could upregulate MMP-9 to compensate for the loss of MMP-2 activity. This is a common phenomenon in which cells modulate the levels of specific MMPs to adapt to environmental changes or maintain their invasive capabilities (Sternlicht and Werb, 2001). It is also possible that MMP-9 regulation is somewhat independent of MMP-2 in our model. While these enzymes can interact, they can also be regulated by different signals. The cellular machinery may respond to TIMP-2-loaded EVs by adjusting MMP-9 expression independently of MMP-2 levels. The interaction between TIMP-2 and MMP-9 might be more complex than simply a one-to-one inhibition, and other intracellular pathways might be at play that influence MMP-9 expression based on the concentration of TIMP-2 (Sternlicht and Werb, 2001). In contrast, the interaction between TIMP-2 and MMP-1 has been studied less extensively than its interactions with MMP-2 or MMP-9, but available evidence suggests that TIMP-2 can effectively inhibit MMP-1 activity (Bahudhanapati et al., 2011). MMP-1, also known as interstitial collagenase, is primarily responsible for the cleavage of fibrillar collagens, such as type I, II, and III collagens, which are significant components of the extracellular matrix (Almutairi et al., 2023). TIMP-2 binds to the active site of MMP-1 in a 1:1 stoichiometric ratio, forming a noncovalent complex that blocks the enzyme’s catalytic activity and prevents collagen degradation (Bahudhanapati et al., 2011). This inhibitory interaction helps maintain the structural integrity of the extracellular matrix and limits excessive tissue remodeling.

In our study, MMP-1 levels did not show significant changes upon treatment with pTIMP-2–loaded EVs. MMP-1 (interstitial collagenase) primarily cleaves fibrillar collagens, contributing to extracellular matrix (ECM) remodeling (Brinckerhoff and Matrisian, 2002). In the context of lung cancer, MMP-1 has been implicated in tumor invasion and metastasis, particularly through facilitating stromal degradation and creating paths for migrating cancer cells (Selvaraj et al., 2019). The observed stability of MMP-1 in our system suggests that the anti-invasive and potential anti-angiogenic effects mediated by pTIMP-2–loaded EVs are largely driven by selective modulation of MMP-2/9 activity, rather than a broad suppression of all collagenases (Stetler-Stevenson, 2008a; Waszczykowska et al., 2020). This specificity is consistent with TIMP-2’s known substrate preference and may indicate a focused therapeutic effect, minimizing off-target disruption of ECM remodeling processes that could otherwise impact normal tissue homeostasis (Brew and Nagase, 2010). Moreover, the lack of MMP-1 alteration supports the concept that TIMP-2–mediated interventions may preferentially inhibit angiogenesis through pathways dominated by MMP-2/9 activity, such as basement membrane degradation and VEGF mobilization, rather than via mechanisms involving MMP-1 (Quintero-Fabián et al., 2019). Overall, while MMP-1 remains an important contributor to tumor invasion in certain contexts, its unchanged levels in our model highlight the specificity of TIMP-2 effects and reinforce the relevance of targeting MMP-2/9 for anti-angiogenic and anti-invasive strategies in lung cancer.

Based on our research, it is essential to consider the potential implications of these findings for targeted gene delivery. While the results suggest that TIMP-2-loaded EVs can modulate MMP-2 expression, further research is needed to confirm the long-term effects and the precise mechanisms by which TIMP-2 regulates MMPs. Additionally, the lack of changes in MMP-1 levels may warrant further exploration of whether TIMP-2-loaded EVs have a broader effect on other members of the MMP family or on other pathways involved in cancer cell migration and invasion. The presence of MMP-2 in our experiment is highly relevant to understanding the regulation of MMP-9 by TIMP-2. The activities of these two MMPs are likely interconnected, and their regulation by TIMP-2-loaded EVs might not follow a straightforward linear relationship. Instead, the cells could be compensating for MMP-2 inhibition by upregulating MMP-9, either directly or through feedback mechanisms. This complex interplay between MMP-2 and MMP-9 underscores the importance of considering multiple factors when studying the regulation of these enzymes in disease contexts. Further investigation into the interplay between these two MMPs and their regulation by TIMP-2 would provide deeper insight into how this balance affects ECM remodeling, especially in cancer cells. Exploring whether MMP-2 presence leads to transcriptional or post-transcriptional changes in MMP-9 expression could further clarify the relationship.

## 4 Conclusion

The potential of extracellular vesicles as carriers of this type of genetic material has generated significant research interest in their roles in gene therapy, cancer treatment, immunotherapy, and regenerative medicine. The use of electroporation to exogenously load TIMP-2 into extracellular vesicles confirmed its effectiveness, despite the low yield. It was shown that pTIMP-2-loaded EVs do not globally affect the balance of all studied metalloproteinases in lung cancer cells. However, the inhibition of MMP-2 expression by TIPM-2-loaded extracellular vesicles indicates a promising direction for the exploration of novel therapeutic targets in lung cancer. Further studies will be necessary to understand the scope and mechanism of this effect fully.

## Data Availability

The raw data supporting the conclusions of this article will be made available by the authors, without undue reservation.
